# Adaptive EWMA control chart using Bayesian approach under ranked set sampling schemes with application to Hard Bake process

**DOI:** 10.1038/s41598-023-36469-7

**Published:** 2023-06-10

**Authors:** Imad Khan, Muhammad Noor-ul-Amin, Dost Muhammad Khan, Salman A. AlQahtani, Wojciech Sumelka

**Affiliations:** 1 Department of Statistics, Abdul Wali Khan University Mardan, Khyber Pakhtunkhwa, Pakistan; 2 Department of Statistics, COMSATS University Islamabad, Lahore Campus, Lahore, Pakistan; 3 Department of Computer Engineering, College of Computer and Information Sciences, King Saud University, P.O. Box 51178, Riyadh, Saudi Arabia; 4 Institute of Structural Analysis, Poznan University of Technology, Poznan, Poland

**Keywords:** Engineering, Mathematics and computing

## Abstract

The memory-type control charts, such as cumulative sum (CUSUM) and exponentially weighted moving average control chart, are more desirable for detecting a small or moderate shift in the production process of a location parameter. In this article, a novel Bayesian adaptive EWMA (AEWMA) control chat utilizing ranked set sampling (RSS) designs is proposed under two different loss functions, i.e., square error loss function (SELF) and linex loss function (LLF), and with informative prior distribution to monitor the mean shift of the normally distributed process. The extensive Monte Carlo simulation method is used to check the performance of the suggested Bayesian-AEWMA control chart using RSS schemes. The effectiveness of the proposed AEWMA control chart is evaluated through the average run length (ARL) and standard deviation of run length (SDRL). The results indicate that the proposed Bayesian control chart applying RSS schemes is more sensitive in detecting mean shifts than the existing Bayesian AEWAM control chart based on simple random sampling (SRS). Finally, to demonstrate the effectiveness of the proposed Bayesian-AEWMA control chart under different RSS schemes, we present a numerical example involving the hard-bake process in semiconductor fabrication. Our results show that the Bayesian-AEWMA control chart using RSS schemes outperforms the EWMA and AEWMA control charts utilizing the Bayesian approach under simple random sampling in detecting out-of-control signals.

## Introduction

Statistical process control (SPC) is considered as an effective statistical technique that are applied to track and manage variations in the production process, ensuring that any deviations from the norm are identified and addressed before they result in production of a defective item. Control charts (*CC*s) play a critical role in SPC as they are the primary tool for monitor the product manufacturing process. The primary aim of the *CC*s is to monitor and detect inconsistencies in the manufacturing process. This process ultimately stability and enhances the overall quality of the final product Montgomery^1^. Shewhart^2^ introduced the memory-less control charting theory concept, which relies solely on current sample information and disregards any prior sample information. The memory-less type *CC*s are effective in detecting significant variations in the manufacturing process; however, they may not be sensitive enough to detect small or moderate shifts. Thus, to identify small to moderate shifts, Page^3^ and Roberts^4^ have investigated the use of cumulative sum (CUSUM) and exponentially weighted moving average (EWMA) *CC*s. These particular *CC*s are recognized for their ability to exhibit memory characteristics, enabling them to consider not only the present but also previous samples of information. Many authors such as Lowry et al.^5^, Lucas and Saccucci^6^, and Zhao et al.^7^ have studied the performance of the EWMA *CC* for detecting small to moderate modifications in the production process. Noor-ul-Amin et al.^8^ and Riaz et al.^9^ have recently conducted studies on modifying the EWMA *CC* to detect small to moderate shifts more effectively. When the process mean experiences a shift, the CUSUM and EWMA *CC*s effectively detect the shift if the size of the shift is known beforehand or if the quality engineer can create a specific shift. However, in situations where the shift size is unknown, an adaptive version of the *CC* provides an advantage by improving detection for varying shift sizes. Capizzi and Masarotto^10^ proposed the adaptive EWMA *CC* to monitor fluctuations in the location parameter of process, by combining EWMA and Shewhart *CC*s. They show that the AEWMA *CC* can monitor the minor, moderate and large shifts in the production process more efficiently than optimal CUSUM, optimal EWMA, and classical Shewhart and EWMA *CC*s. Adaptive cumulative sum (ACUSUM) *CC* for supervising process mean was studied by Sparks^11^. This method can detect displacements of different magnitudes faster than the conventional CUSUM *CC*. Zaman et al.^12^ suggested a novel adaptive EWMA control design that utilizes the Tukey bi-square function to monitor the process mean of a location parameter. The effectiveness of this approach was assessed through various performance metrics, including ARL, extra quadratic loss, and relative ARL. The authors, such as Jiang et al.^13^, Wu et al.^14^, Huang et al.^15^, Aly et al.^16^ and Aly et al.^17^ worked on the AEWMA and ACUSUM *CC*s for monitoring variations in the production process and stability of the final product. Haq et al.^18^ studied adaptive EWMA *CC* that utilizes EWMA statistic to detect variations in the location parameter of the process. Their study found that, their suggested *CC* outperformed other popular *CC* methods, including Shewhart cumulative sum (SCUSUM), double exponentially weighted moving (DEWMA), classical CUSUM, and classical EWMA *CC*s, in terms of both the ARL and the SDRL performance measures.

All these studies have been conducted to improve the classical approach; this describes a method that solely depends on the information from the current sample, disregarding any prior knowledge. The Bayesian estimation method combines both the sample and prior information that update the posterior distribution used for estimating of the unknown population mean. Girshick and Rubin^19^ first who suggested the Bayesian *CC*s. Menzefricke^20^ proposed a *CC* for monitoring the process mean of the location parameter based on Bayesian theory. Abbas et al.^21^ suggested DEWMA *CC* using the Bayesian approach, which can monitor the process mean with dissimilar shift size more quickly than the classical EWMA *CC*. The Bayesian *CC* using different symmetric and asymmetric loss functions (LFs) is studied by Ali and Riaz^22^. Asalam et al.^23^ suggested the improved Bayesian modified-EWMA *CC* applying posterior (P) and posterior predictive (PP) distribution, and to assess the performance of the proposed *CC*, both ARL as well as SDRL are employed as evaluation metrics. The findings advocate that the modified Bayesian-EWMA *CC* is more effective than the current *CC* in detecting out-of-control signals. Noor et al.^24^ developed the Bayesian-EWMA *CC* by incorporating both exponential and transformed exponential distributions and employing various LFs. Furthermore, they investigated the influence of the loss function selection on the performance of the Bayesian-EWMA *CC*. Lin et al.^25^ developed the Bayesian-EWMA *CC* for monitoring the variance of a distribution-free process. The proposed statistic was assessed for its sampling properties, making it well-suited for monitoring fluctuations in the time-varying process distribution. Moreover, a simulation study provides evidence of the efficacy of the *CC*. Khan et al.^26^ proposed a novel approach based Hybridized Bayesian EWMA *CC* for monitoring the location parameter using various RSS schemes and an informative prior. The authors evaluated the *CC* performance using ARL and SDRL and compared it with other Bayesian control charts, such as HEWMA and AEWMA with Bayesian theory, under SRS. Ranked-based Sampling designs is a sampling strategy that is commonly utilized in quality control applications to reduce the expense of data collection. This method involves ranking the units in each group and selecting a subset of ranked units as the sample. By utilizing RSS, the efficiency and accuracy of the *CC* can be improved. Moreover, the combination of RSS with the Bayesian approach and AEWMA *CC* can further enhance the performance of the *CC*. This combined approach leverages the efficient sampling method of RSS and the flexibility of Bayesian updating to adjust the control limits based on the available data. Combining RSS with the Bayesian approach in AEWMA *CC* can prove highly advantageous in scenarios where data collection costs are high or the sample size is limited. In this regard, we suggested Bayesian-AEWMA *CC* that employs three distinct ranked set sampling schemes: ranked set sampling (RSS), median ranked set sampling (MRSS), and finally extreme ranked set sampling (ERSS) based on informative prior applying two various LFs P and PP distributions. The proposed Bayesian AEWMA *CC*s efficacy was assessed by measuring its ARL and SDRL.

The remaining article is structured into several sections. Section "Bayesian approach" provides a brief introduction to Bayesian theory and LFs. In Section "Ranked set sampling", we discuss various RSS schemes. Section "Proposed Bayesian AEWMA CC using various LFS under RSS designs" describes a Bayesian-AEWMA CC under various RSS schemes. Section "Simulation study" contains the simulation steps and discussion and the main findings are included in section "Discussion and main findings". Section "Real-life applications" presents a numerical analysis, while the conclusion is provided in section "Conclusion".

## Bayesian approach

In statistical inference, the classical and Bayesian approaches are the two estimation methods. The classical method of estimation is based on only sample information while in the Bayesian approach both types of information, i.e. prior and sample information, are utilized when estimating the unknown population parameter. The Bayesian approach is founded on the concept that probability can represent uncertainty, and permits the incorporation of prior knowledge and uncertainty into our analysis. Bayesian inference requires the use of a prior distribution, which means that both non-informative and informative priors are relevant to Bayesian analysis. On the other hand, a conjugate prior refers to a situation where the prior distribution and the sampling distribution belong to the same family of distributions. It is widely used in machine learning, finance, and medical research, where incomplete information and uncertainty are standard. In this study, we define the variable *X* as the study characteristic with an in-control process mean of $\theta$ and a variance of $\delta^{2}$ and taking normal conjugate prior under parameters $\theta_{0}$ and $\delta_{0}^{2}$, is mathematized as:

1 $$p\left( \theta \right) = \frac{1}{{\sqrt {2\pi \delta_{0}^{2} } }}\exp \left\{ { - \frac{1}{{2\delta_{0}^{2} }}\left( {\theta - \theta_{0} } \right)^{2} } \right\}.$$

But if there is a lack of information regarding the population parameter, the prior is considered non-informative, which has a minimal impact on the P distribution, reflecting the Bayesian approach to incorporating prior information into the analysis. In many cases, a non-informative prior is assumed to be proportional to a uniform distribution, which assigns equal probability to all possible parameter values within a specified range. The uniform prior distribution is represented by the probability function given as:

2 $$p\left( \theta \right) \propto \sqrt {\frac{n}{{\delta^{2} }}} = c\sqrt {\frac{n}{{\delta^{2} }}} ,$$

where *c* represents a constant of proportionality.

When little or no prior information is available for an unknown population parameter, a non-informative prior is commonly used in Bayesian analysis. This type of prior distribution has a minimal effect on the posterior distribution. Jeffrey^27^ proposed a prior distribution that is proportional to the Fisher information matrix in such situations, the $p\left( \theta \right)$ is mathematized as

3 $$p\left( \theta \right) \propto \sqrt {I\left( \theta \right)} .$$

In Eq. (3), $I\left( \theta \right) = - E\left( {\frac{{\partial^{2} }}{{\partial \theta^{2} }}\log f\left( {X/\theta } \right)} \right)$ shows the Fisher information matrix and it allows for the incorporation of available information on the parameter into the analysis.

Bayesian statistics relies on integrating sample information based a prior distribution to obtain a P distribution that encompasses all relevant knowledge about the unknown population parameter. The P distribution is updated and provides more information than the prior distribution, as it incorporates both the sample and prior information to produce a probability distribution for the given parameter $\theta$ is defined as

4 $$p\left( {\theta |x} \right) = \frac{{p\left( {x|\theta } \right)p\left( \theta \right)}}{{\int {p\left( {x|\theta } \right)p\left( \theta \right)d\theta } }}.$$

The PP distribution using P distribution as a prior distribution for novel data-set *Y* follows

5 $$p(y|x) = \int {p(y|\theta )p(\theta |x)d\theta } .$$

In Bayesian methodology, the LF choice is pivotal in reducing the risks associated with the Bayes estimator. In this study, we examine two distinct LFs.

### Squared error loss function

Gauss^28^ illustrates a symmetric loss function called SELF that can be used in statistical estimation. If we have an estimator $\hat{\theta }$ for a population parameter (unknown) $\theta$, then SELF can be mathematized as:

6 $$L\left( {\theta ,\hat{\theta }} \right) = \left( {\theta - \hat{\theta }} \right)^{2} ,$$

Bayes estimator using SELF is $\hat{\theta } = E_{\theta /x} \left( \theta \right)$.

### Linex loss function

Varian^29^ conducted a study on a specific type of loss function called the Linex Loss Function. This LFs is asymmetric and is used to minimize risks connected with the Bayesian estimator. The LLF is defined as follows:

7 $$L\left( {\theta ,\hat{\theta }} \right) = \left( {e^{{c\left( {\theta - \hat{\theta }} \right)}} - c\left( {\theta - \hat{\theta }} \right) - 1} \right),$$

Based on LLF, $\hat{\theta }$ which shows the Bayesian estimator and is given by $\hat{\theta } = - \frac{1}{c}InE_{\theta /x} \left( {e^{ - c\theta } } \right)$.

## Ranked set sampling

The notion of RSS was initially presented by McIntyre^30^. This technique involves the following steps for selecting a sample from the population of interest:

1. Select *m*^2^ random samples independently from the studied population and distributed into *m* sets with equal size. Array all *m* units by the personal judgment of the researcher or by using auxiliary variables and/or any numerical method without measurement.
2. After ordering all the *m* sets, select 1^st^ unit from the first set and 2^nd^ unit is chosen from the second set and so on. Completing the steps outlined above results in a single cycle of RSS. if required, one can repeat these steps *r* times, resulting in a sample size of $n = rm$

Under the RSS scheme, the estimator for population mean with a single cycle is mathematized as

8 $$\overline{Z}_{{\left( {RSS} \right)}} = \frac{1}{m}\sum\limits_{i = 1}^{m} {Z_{i\left( i \right)} } ,$$

and

9 $${\text{var}} \left( {\overline{Z}_{{\left( {RSS} \right)}} } \right) = \frac{{\delta^{2} }}{m} - \frac{1}{{m^{2} }}\sum\limits_{i = 1}^{m} {\left( {\mu_{\left( i \right)} - \mu } \right)} .$$

### Median ranked set sampling

Muttlak^31^ introduced a revised RSS method known as the well-known Median Ranked Set Sampling (MRSS) scheme. The estimator under the MRSS scheme more efficiently estimates the population mean. The following steps provide a detailed overview of the procedure for selecting a sample using the MRSS technique. Commonly used in survey research, this technique can help ensure accurate estimates while minimizing costs and resources:

1. To apply the MRSS scheme, Choose the $m^{2}$ units from the population under study using the same method as for RSS. Next, distributed the selected elements into *m* similar sets and rank the elements within each set in ascending order based on the variable of interest.
2. If the set size is even, select the smallest element from the middle two elements (*m*/2)th element and the largest elements from the middle two (*m*/2)th sampling elements. In case the *m* is odd, select median elements from ((*m* + 1)/2)th arranged sets. This process completes a single cycle of MRSS. If necessary, replicate the process *r* times to obtain a desired sample size $n = rm$.

Utilizing MRSS, the population mean estimator for odd sample size under a single cycle is mathematized as

10 $$\overline{Z}_{{\left( {MRSS} \right)O}} = \frac{1}{m}\left( {\sum\limits_{i = 1}^{m} {Z_{{i\left( {\frac{m + 1}{2}} \right)}} } } \right),$$

and

11 $${\text{var}} \left( {\overline{Z}_{{\left( {MRSS} \right)O}} } \right) = \frac{1}{m}\left( {\delta_{{\left( {\frac{m + 1}{2}} \right)}}^{2} } \right).$$

For a single cycle of MRSS, the population mean estimator for the case even sample size is given as::

12 $$\overline{Z}_{{\left( {MRSS} \right)E}} = \frac{1}{m}\left( {\sum\limits_{i = 1}^{m/2} {Z_{{i\left( \frac{m}{2} \right)}} } + \sum\limits_{i = 1}^{m/2} {Z_{{\frac{m}{2} + i\left( {\frac{m + 1}{2}} \right)}} } } \right),$$

with variance

13 $${\text{var}} \left( {\overline{Z}_{{\left( {MRSS} \right)E}} } \right) = \frac{1}{m}\left( {\delta_{{\left( \frac{m}{2} \right)}}^{2} + \delta_{{\left( {\frac{m + 2}{2}} \right)}}^{2} } \right).$$

### Extreme ranked set sampling apporoach

A modified ranked set sampling approach, known as the extreme ranked set sampling (ERSS) scheme, was proposed by Amawi et al.^32^. This method is particularly useful when collecting extreme elements is difficult. The following steps provide a detailed overview of the process for selecting an ERSS sample.

1. The $m^{2}$ units are selected from the population under study and allocated randomly in *m* sets of equal size, assigning ranks to each unit within a set based on a particular study variable.
2. For ERSS, the selection of units depends on the sample size along with the number of order sets. In case even sample size, then both the smallest and largest elements should be chosen from the first and last order sets corresponding to the middle half of the ranked units, i.e. $(m/2)$th. In contrast, if the sample size is odd, the smallest and largest elements should be selected from the first and last order sets that correspond to the outer halves of the ranked units i.e., $(m - 1/2)th$, and the median element should be chosen from the last order set.

If essential, the complete method of ERSS is repeated *r* time to get the required sample size *n* = *mr*. For odd sample size, under ERSS the mean estimator for the population mean (unknown) using ERSS using single cycle can be written as:

14 $$\overline{Z}_{{\left( {ERSS} \right)O}} = \frac{1}{m}\left( {\sum\limits_{i = 1}^{{\left( {\frac{m - 1}{2}} \right)}} {Z_{i\left( 1 \right)} } + \sum\limits_{i = 1}^{{\left( {\frac{m - 1}{2}} \right)}} {Z_{{\left( {\frac{m - 1}{2}} \right) + i\left( l \right)}} } + Z_{{m\left( {\frac{m + 1}{2}} \right)}} } \right),$$

with

15 $${\text{var}} (\overline{Z}_{{\left( {ERSS} \right)O}} ) = \frac{1}{{2m^{2} }}\left( {\delta_{\left( 1 \right)}^{2} + \delta_{\left( m \right)}^{2} } \right) + \frac{1}{{l^{2} }}\left( {\delta_{{\left( {\frac{m + 1}{2}} \right)}}^{2} } \right).$$

Utilizing ERSS with an even sample size, the mean estimator utilizing single cycle is follows as:

16 $$\overline{Z}_{{\left( {ERSS} \right)E}} = \frac{1}{m}\left( {\sum\limits_{i = 1}^{{\left( \frac{m}{2} \right)}} {Z_{i\left( 1 \right)} } + \sum\limits_{i = 1}^{{\left( \frac{m}{2} \right)}} {Z_{{\frac{m}{2} + i\left( l \right)}} } } \right),$$

and

17 $${\text{var}} \left( {\overline{Z}_{{\left( {ERSS} \right)E}} } \right) = \frac{1}{2m}\left( {\delta_{\left( 1 \right)}^{2} + \delta_{\left( m \right)}^{2} } \right).$$

## Proposed Bayesian AEWMA *CC* using various LFS under RSS designs

This section describes the suggested AEWMA *CC* for monitoring variation and detecting the small, moderate and large shifts of a normally distributed manufacturing process using different RSS schemes. Consider the study variable *X* that following a normal distribution with $\theta$ and $\delta^{2}$ as a mean and variance respectively. The probability function for *X* can be expressed as

18 $$f\left( {x_{t} :\theta ,\delta^{2} } \right) = \frac{1}{{\sqrt {2\pi \delta^{2} } }}\exp \left( { - \tfrac{1}{{2\delta^{2} }}\left( {x_{t} - \theta } \right)^{2} } \right).$$

Let the $\widehat{{\delta_{t}^{*} }}$ be the sequence of EWMA statistic applying $\left\{ {X_{t} } \right\}$, given by:

19 $$\widehat{{\delta_{t}^{*} }} = \psi X_{t} + \left( {1 - \psi } \right)\widehat{{\delta_{t - 1}^{*} }},$$

where $\psi$ is a smoothing constant and $\widehat{{\delta_{0}^{*} }} = 0$. The estimator $\widehat{{\delta_{t}^{*} }}$ is unbiased for the in-control process and biased for the out-of-control process. Haq et al.^18^ proposed an unbiased estimator $\delta$ for both the case of in-control and out-of-control situations, which is given by

20 $$\widehat{{\delta_{t}^{**} }} = \frac{{\widehat{{\delta_{t}^{*} }}}}{{1 - \left( {1 - \psi } \right)^{t} }},$$

They suggested to use $$\widehat{{\delta_{t} }} = |\widehat{{\delta_{t}^{**} }}|$$, to estimate $$\delta$$.

The proposed AEWMA *CC* under Bayesian theory applying various ranked-based sampling designs for the process mean using the sequence $\hat{\theta }_{{\left( {RSS_{i} } \right)LF}}$ is given by

21 $$W_{t} = v\left( {\hat{\delta }_{t} } \right)\hat{\theta }_{{\left( {RSS_{i} } \right)LF}} + \left( {1 - v\left( {\hat{\delta }_{t} } \right)} \right)W_{t - 1} ,$$

Such that *i* = 1, 2, 3. $$\begin{gathered} RSS_{1} = RSS \hfill \\ RSS_{2} = MRSS \hfill \\ RSS_{3} = ERSS \hfill \\ \end{gathered}$$, $v\left( {\hat{\delta }_{t} } \right) \in \left( {0,\left. 1 \right]} \right.$ and $W_{0} = 0$ such that

22 $$v({\widehat{\delta }}_{t})=\left\{\begin{array}{l}0.015, if 0.00<{\widehat{\delta }}_{t}\le 0.25\\ 0.10 , if 0.25<{\widehat{\delta }}_{t}\le 0.75\\ 0.20, if 0.75<{\widehat{\delta }}_{t}\le 1.00\\ 0.25, if 1.00<{\widehat{\delta }}_{t}\le 1.50\\ 0.50, if 1.50<{\widehat{\delta }}_{t}\le 2.50 \\ 0.80, if 2.50<{\widehat{\delta }}_{t}\le 3.50\\ { 1 , if \widehat{\delta }}_{t}>3.50.\end{array}\right.$$

In case, if the plotting statistic cross the threshold value $h$ then the process is said to be out-of-control otherwise the process is in control.

In case the prior and sampling distribution are both normal distributions than P distribution will also be a normal distribution. The mean and standard deviation of the P distribution are given by $\theta_{n}$ and $\delta_{n}$ respectively. The $P\left( {\theta /x} \right)$ can be demonstrated as follows:

23 $$P\left( {\theta /x} \right) = \frac{1}{{\sqrt {2\pi } \sqrt {\frac{{\delta^{2} \delta_{0}^{2} }}{{\delta^{2} + n\delta_{0}^{2} }}} }}\exp \left[ { - \frac{1}{2}\left( {\frac{{\theta - \sum\limits_{i = 1}^{n} {\frac{{x_{i} \delta_{0}^{2} + \theta_{0} \delta_{0}^{2} }}{{\delta^{2} + n\delta_{0}^{2} }}} }}{{\sqrt {\frac{{\delta^{2} \delta_{0}^{2} }}{{\delta^{2} + n\delta_{0}^{2} }}} }}} \right)} \right],$$

where $\theta_{n} = \frac{{n\overline{x} \delta_{0}^{2} + \delta^{2} \theta_{0} }}{{\delta^{2} + n\delta_{0}^{2} }}$ and $\delta_{n} = \sqrt {\frac{{\delta^{2} \delta_{0}^{2} }}{{\delta^{2} + n\delta_{0}^{2} }}}$ respectively.

Under SELF, the Bayes estimator using various RSS schemes is mathematized as

24 $$\hat{\theta }_{{RSS_{i} \left( {SELF} \right)}} = \frac{{n\overline{x}_{{(RSS_{i} )}} \delta_{0}^{2} + \delta^{2} \theta_{0} }}{{\delta^{2} + n\delta_{0}^{2} }}.$$

The properties of the $\hat{\theta }_{{\left( {SELF} \right)}}$ is given as

$E\left( {\hat{\theta }_{{\left( {SELF} \right)}} } \right) = \frac{{n\theta_{1} \delta_{0}^{2} + \delta^{2} \theta_{0} }}{{\delta^{2} + n\delta_{0}^{2} }}$ and $sd\left( {\hat{\theta }_{{\left( {SELF} \right)}} } \right) = \sqrt {\frac{{n\delta_{{(RSS_{i} )}}^{2} \delta_{0}^{4} }}{{\delta^{2} + n\delta_{0}^{2} }}}$ respectively. The Bayes estimator $\hat{\theta }_{{RSS_{i} \left( {_{LLF} } \right)}}$ for suggested Bayesian *CC* using LLF applying ranked-based sampling methods is derived as

25 $$\hat{\theta }_{{RSS_{i} \left( {_{LLF} } \right)}} = \frac{{n\overline{x}_{{(RSS_{i} )}} \delta_{0}^{2} + \delta^{2} \theta_{0} }}{{\delta^{2} + n\delta_{0}^{2} }} - \frac{{C^{^{\prime}} }}{2}\delta_{n}^{2} .$$

The mean and standard deviation of $\hat{\theta }_{{\left( {_{LLF} } \right)}}$ is given by

$$E\left( {\hat{\theta }_{LLF} } \right) = \frac{{n\theta_{1} \delta_{0}^{2} + \delta^{2} \theta_{0} }}{{\delta^{2} + n\delta_{0}^{2} }} - \frac{{C^{^{\prime}} }}{2}$$ and $$sd\left( {\hat{\theta }_{LLF} } \right) = \sqrt {\frac{{n\delta_{{(RSS_{i} )}}^{2} \delta_{0}^{4} }}{{\left( {\delta^{2} + n\delta_{0}^{2} } \right)^{2} }}}$$ respectively.

The suggested Bayesian-AEWMA CC, which utilizes various RSS schemes for the P and PP distributions, is defined based on a set of feature observations of size *h* denoted as $y_{1} ,y_{2} ,....,y_{h}$ is given by

26 $$p\left( \frac{y}{x} \right) = \frac{1}{{\sqrt {2\pi \delta_{1}^{2} } }}\exp \left\{ { - \frac{1}{{2\delta_{1}^{2} }}\left( {Y - \theta_{n} } \right)^{2} } \right\},$$

which is normally distributed with mean and variance $\theta_{n}$ and $\delta_{1}$ respectively, derived as $\delta_{1} = \sqrt {\delta^{2} + \frac{{\delta^{2} \delta_{0}^{2} }}{{\delta^{2} + n\delta_{0}^{2} }}}$. The estimator $\hat{\theta }$ for PP distribution utilizing LLF under different RSS schemes is defined as

27 $$\hat{\theta }_{{RSS_{i} (LLF)}} = \frac{{n\overline{x}_{{(RSS_{i} )}} \delta_{0}^{2} + \delta^{2} \theta_{0} }}{{\delta^{2} + n\delta_{0}^{2} }} - \frac{{C^{^{\prime}} }}{2}\tilde{\delta }_{1}^{2} ,$$

where $\tilde{\delta }_{1}^{2} = \frac{{\delta^{2} }}{k} + \frac{{\delta^{2} \delta_{0}^{2} }}{{\delta^{2} + n\delta_{0}^{2} }}$. The mean and standard deviation of $\hat{\theta }_{LLF}$ is given as

$$E\left( {\hat{\theta }_{LLF} } \right) = \frac{{n\theta_{1} \delta_{0}^{2} + \delta^{2} \theta_{0} }}{{\delta^{2} + n\delta_{0}^{2} }} - \frac{{C^{^{\prime}} }}{2}\tilde{\delta }_{1}^{2}$$ and $$sd\left( {\hat{\theta }_{LLF} } \right) = \sqrt {\frac{{n\delta_{{(RSS_{i} )}}^{2} \delta_{0}^{4} }}{{\left( {\delta^{2} + n\delta_{0}^{2} } \right)^{2} }}}$$ respectively.

## Simulation study

To appraise the efficacy of the suggested AEWMA *CC* with different ranked-based designs applying the Bayesian approach under an informative prior distribution using the Monte Carlo simulation technique. We take two different values of smoothing constants *i.e.,* $\psi = 0.10$ and $\psi = 0.25$ to study the effectiveness of smoothing constants on the suggested Bayesian-AEWMA *CC*. The complete simulation steps follow as:

### Estimating the threshold for an in-control ARL

i. When calculating the mean as well as variance of both the P distribution and PP distribution under different LFs, we employed the standard normal distribution as prior and sampling distribution. *i.e.,* $E\left( {\hat{\theta }_{{\left( {RSS_{i} } \right)LF}} } \right)$ and $\delta_{{\left( {RSS_{i} } \right)LF}}$.
ii. For an unchangeable value of smoothing constant $\psi$ choose a value of *h*.
iii. From normal distribution, select a ranked set sample of size *n* for in-control process i.e., $X \sim N\left( {E\left( {\hat{\theta }} \right),\delta^{2} } \right)$.
iv. Compute the suggested Bayesian-AEWMA statistic given in Eq. (21), and evaluate the process accordingly.
v. If the process is determined to be in-control, continue with the steps above until an out-of-control signal is observed, and make a record of the number of consecutive in-control run lengths.

### Setting the threshold for out-of-control ARL

i. Create a random sample drawn from a normal distribution, but with a mean that has been shifted. i.e., $X \sim N(E(\hat{\theta }_{LF} ) + \delta \frac{\sigma }{\sqrt n },\delta )$.
ii. Calculate $W_{t}$ and assess the procedure using the proposed AEWMA *CC* under the Bayesian approach applying various RSS designs.
iii. If the process is deemed to be in control, the two steps mentioned earlier should be repeated until the process is declared out-of-control. It is also essential to keep track of the number of in-control runs for record-keeping purposes.
iv. Execute the previously described steps repeatedly for 100,000 iterations to determine the run-length profiles.

## Discussion and main findings

**Figure 1:**
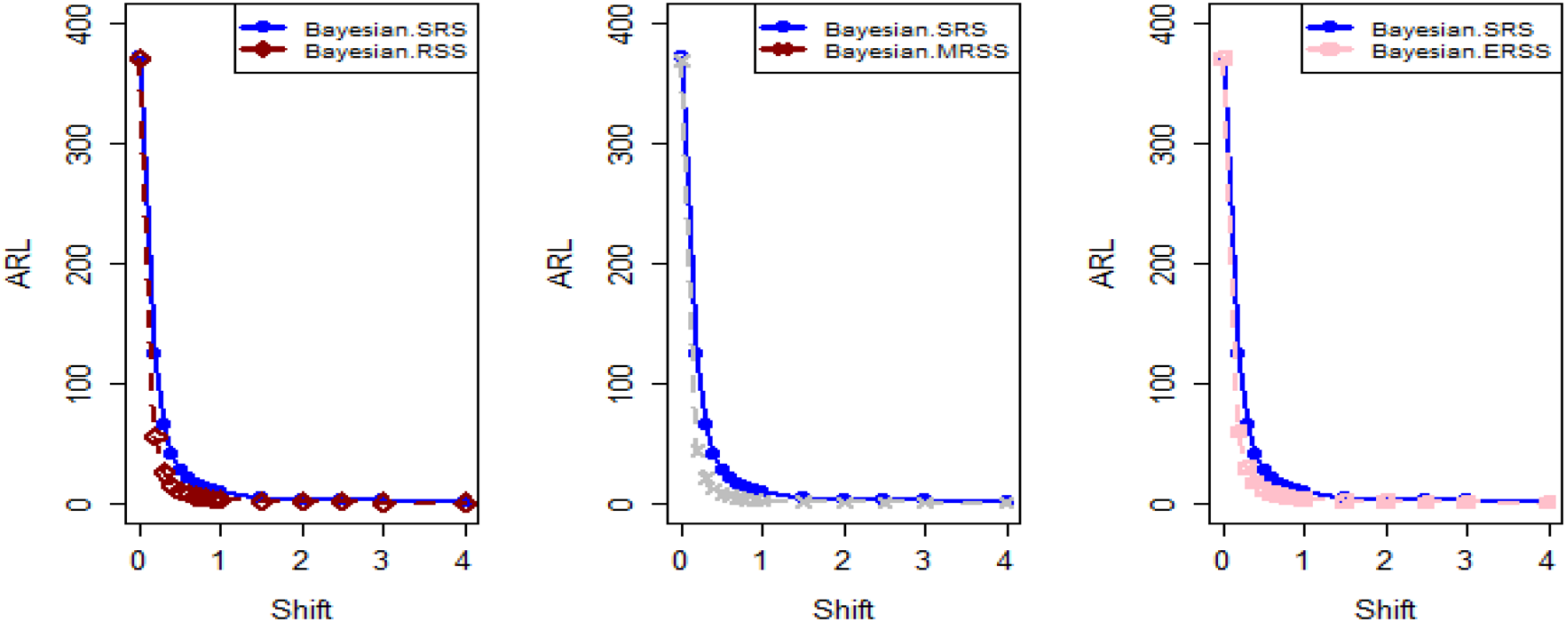
Under SELF, ARL plots using P and PP distribution with different ranked-based sampling schemes.

**Figure 2:**
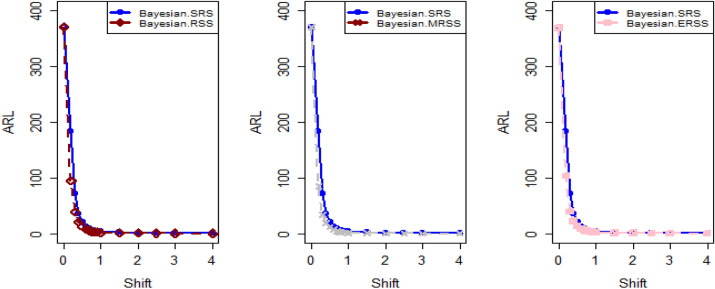
Utilizing LLF, ARL plots of suggested AEWMA *CC* under RSS, MRSS, and ERSS.

**Figure 3:**
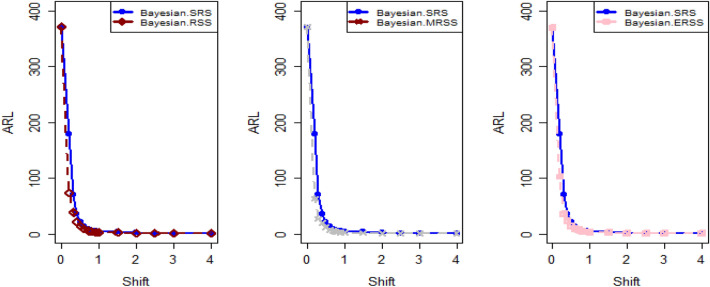
Under LLF, ARL plots using PP distribution applying distinct RSS schemes.

Tables 1, 2, 3, 4, 5 and 6 display a comparison between the Bayesian-AEWMA *CC* under SRS and the proposed Bayesian *CC* based on various RSS designs under an informative prior. The comparison is performed using two distinct LFs at different values of the smoothing constant $\psi$. The outcomes presented in Tables 1 and 2 indicate the effectiveness of the EWMA and adaptive EWMA *CC* applying Bayesian approach using SRS, and proposed *CC* utilizing RSS, MRSS, and ERSS schemes for P and PP distribution based on SELF. The findings demonstrate that the proposed *CC* utilizing RSS schemes is more efficient in detecting out-of-control signals compared to the existing EWMA and AEWMA *CC* under Bayesian methodology utilizing SRS. *i.e.,* the ARL outcomes of the EWMA *CC* under Bayesian theory*,* using SELF at smoothing constant $\psi = 0.10$ and different shifts *i.e.,* $\sigma$ = 0.0, 0.30, 0.50, 0.80, 1.50, 4 are 370.63, 115.00, 28.51, 13.38, 5.82 and 2.13, and for Bayesian-AEWMA *CC* the ARL results are 370.16, 35.40, 13.55, 5.62, 2.25 and 1.02. Utilizing analogous condition ARL outcomes of proposed AEWMA *CC* applying RSS, MRSS and ERSS are 370.25, 20.53, 8.87, 3.07, 1.26, 1 and the values under MRSS are 371.55, 19.83, 8.34, 2.86, 1.18, 1 and 370.13, 22.38, 9.52, 3.36, 1.37 and 1 are the $ARL$ values for ERSS. In the same way, Table 6 shows the comparison of EWMA and AEWMA *CC* with Bayesian approach using SRS with the proposed *CC* using RSS schemes under LLF, the ARL results of Bayesian-EWMA *CC* utilizing SRS at $\psi = 0.25$ and shifts $\sigma$ = 0.0, 0.30, 0.50, 0.80, 1.50, 4 are 370.23, 103.68, 41.26, 15.79, 5.18, and 1.66, and 369.58, 54.92, 25.97, 12.79, 4.94 and 1.09 are the respective numeric of ARL for Bayesian-AEWMA *CC*, for proposed AEWMA *CC* under RSS the $ARL$ values with same smoothing constant and shifts are 371.18, 23.55, 8.64, 3.92, 2.46, 1 for MRSS the $ARL$ values are 370.56, 18.70, 7.15, 2.66, 1.20, 1 and $ARL$ values for the ERSS are 369.23, 30.52, 11.15, 4.45, 2.01, and 1. At the larger shift the $ARL$ values decrease rapidly which shows the superiority of the proposed *CC* applying Bayesian theory with various ranked-based sampling designs, and more quickly detect the out-of-control signals than the existing Bayesian-EWMA and Bayesian-AEWMA *CC*. and the ARL Figs. 1, 2 and 3 also show the efficiency of the proposed Bayesian AEWMA *CC* under RSS schemes. The following are key discoveries of the suggested *CC* using distinct LFs and under various ranked-based sampling designs using P and PP distribution:

- The run length profiles result of suggested AEWMA *CC* Bayesian theory utilizing ranked-based sampling designs using SELF decrease quickly with increases in the mean shift are presented in Tables 1 and 2, which indicate that the proposed Bayesian AEWMA *CC* can quickly detect the shifts in the mean process. For example, at $ARL_{0} = 370$ and $\psi = 0.10$ with shift δ = 0.20 and 0.70, the ARL values are 38.40 and 3.93 for RSS, and for MRSS 32.19 and 3.14 while 39.45 and 3.92 are the ARL results of ERSS.
- The robustness of the suggested AEWMA *CC* based on Bayesian approach applying various RSS designs by using a normal prior distribution with LLF at $\psi = 0.10$ and $0.25$. Tables 3 and 4 present run-length profiles of the proposed AEWMA *CC* based on Bayesian approach, which indicates that the strength of the suggested *CC* is inversely proportional to the values of the smoothing constant. For example, at $ARL_{0} = 370$, $\psi = 0.10$ and shift δ = 0.30, ARL value for the proposed *CC* using RSS is 19.78, for MRSS the ARL value are 15.88 and 21.95 for ERSS. For the mean shift δ = 0.30 and $\psi = 0.25$ the ARL results for using RSS is 29.15, for MRSS 23.39 is the ARL value and the ARL value for the ERSS is 31.99.
- Tables 5 and 6 show the ARL as well as SDRL values for the offered AEWMA *CC* utilizing RSS designs applying LLF based on informative prior using P and PP distribution. The outcomes indicate that the ARL values of offered AEWMA with Bayesian approach *CC* applying RSS at $ARL_{0} = 370$, δ = 0.20 and $\psi = 0.10$ is 39.15, and the ARL value in the same case at $\psi = 0.25$ is 51.42, similarly for MRSS the ARL values is 30.55 and 36.48 and in the similar case, ARL values for ERSS is 38.96 and 54.59.

**Table 1:** Under SELF, the run-length profile based on P and PP distribution for Bayesian-AEWMA *CC*, for $\psi$ = 0.10*, n* = *5.*

Shift	Bayes-EWMA SRS		Bayes-AEWMA SRS		Bayes-AEWMA RSS		Bayes-AEWMA MRSS		Bayes-AEWMA ERSS	
	ARL	SDRL	ARL	SDRL	ARL	SDRL	ARL	SDRL	ARL	SDRL
	L = 2.7047		h = 0.0856		h = 0.0399		h = 0.0366		h = 0.0448	
0.00	370.63	368.13	372.86	537.77	370.42	423.93	370.93	412.73	370.43	473.30
0.20	123.94	115.00	70.61	91.12	36.46	30.79	34.70	26.75	41.78	37.68
0.30	115.00	57.42	35.40	44.53	20.53	17.79	19.83	15.40	22.38	20.29
0.40	41.33	32.49	21.15	26.36	12.99	11.96	12.78	10.68	14.10	13.59
0.50	28.51	20.18	13.55	16.69	8.87	8.69	8.34	7.77	9.52	9.81
0.60	20.95	13.50	9.46	11.23	5.91	6.27	5.58	5.65	6.51	7.03
0.70	16.46	9.64	7.08	7.70	4.17	4.40	3.92	4.06	4.48	4.84
0.75	14.79	8.35	6.15	6.43	3.54	3.71	3.32	3.36	3.83	3.99
0.80	13.38	7.17	5.62	5.82	3.07	3.01	2.86	2.84	3.36	3.33
0.90	11.29	5.57	4.51	4.18	2.51	2.24	2.25	1.93	2.72	2.42
1.00	9.79	4.49	3.85	3.20	2.07	1.60	1.86	1.36	2.24	1.72
1.50	5.82	2.03	2.25	1.29	1.26	0.52	1.18	0.44	1.37	0.60
2.00	4.18	1.20	1.66	0.78	1.06	0.24	1.02	0.16	1.02	0.14
2.50	3.31	0.84	1.36	0.56	1.00	0.08	1	0	1	0.06
3.00	2.75	0.66	1.17	0.39	1	0	1	0	1	0
4.00	2.13	0.383	1.02	0.14	1	0	1	0	1	0

**Table 2:** Under SELF, the run length profile utilizing P and PP distribution for Bayesian-AEWMA *CC* for $\psi$ = 0.25, *n* = *5.*

Shift	Bayes-EWMA SRS		Bayes-AEWMA SRS		Bayes-AEWMA RSS		Bayes-AEWMA MRSS		Bayes-AEWMA ERSS	
	ARL	SDRL	ARL	SDRL	ARL	SDRL	ARL	SDRL	ARL	SDRL
	L = 2.8987		h = 0.241		h = 0.0546		h = 0.03865		h = 0.0758	
0.00	369.49	364.82	369.00	367.39	370.42	428.73	369.06	425.35	371.88	411.70
0.20	178.20	175.14	97.04	80.91	47.08	42.97	33.94	28.30	46.16	42.90
0.30	104.70	100.95	55.71	42.80	23.94	21.86	18.62	15.89	30.39	27.90
0.40	63.11	58.20	36.15	25.09	14.00	13.44	11.37	10.55	17.32	15.91
0.50	41.21	36.61	25.95	17.04	8.66	8.32	7.22	7.15	10.95	9.66
0.60	28.45	24.57	19.80	12.20	5.90	5.42	4.92	4.88	7.62	6.43
0.70	20.61	16.37	15.41	9.09	4.42	3.69	3.50	3.28	5.69	4.37
0.75	17.97	13.87	14.11	8.17	3.87	3.04	3.07	2.72	5.01	3.71
0.80	15.71	11.75	12.87	7.26	3.46	2.60	2.64	2.16	4.41	2.96
0.90	12.51	8.86	10.76	5.97	2.86	1.90	2.19	1.60	3.69	2.29
1.00	10.22	6.77	9.17	4.96	2.43	1.45	1.88	1.20	3.19	1.72
1.50	5.15	2.51	4.90	2.77	1.54	0.64	1.20	0.44	2.01	0.73
2.00	3.46	1.33	2.98	1.83	1.18	0.39	1.03	0.18	1.53	0.55
2.50	2.66	0.86	1.98	1.15	1.04	0.20	1	0	1.22	0.42
3.00	2.19	0.61	1.48	0.72	1	0	1	0	1	0
4.00	1.66	0.50	1	0	1	0	1	0	1	0

**Table 3:** Using LLF, the run length profile using P distribution for Bayesian-AEWMA *CC*, for $\psi$ = 0.10, *n* = *5.*

Shift	Bayes-EWMA SRS		Bayes-AEWMA SRS		Bayes-AEWMA RSS		Bayes-AEWMA MRSS		Bayes-AEWMA ERSS	
	*ARL*	SDRL	ARL	SDRL	ARL	SDRL	ARL	SDRL	ARL	SDRL
	L = 2.7047		h = 0.086		h = 0.04155		h = 0.0367		h = 0.0445	
0.00	370.63	368.13	370.98	539.06	370.22	444.84	370.62	432.08	369.39	478.05
0.20	123.94	115.00	71.98	92.48	38.92	32.65	34.34	26.39	40.25	36.17
0.30	115.00	57.42	36.26	45.49	21.90	18.59	19.71	15.39	22.60	20.23
0.40	41.33	32.49	21.09	26.30	13.89	12.61	12.45	10.70	14.07	13.57
0.50	28.51	20.18	13.71	16.73	8.96	8.96	8.36	7.82	9.38	8.45
0.60	20.95	13.50	9.53	11.25	5.98	6.36	5.66	5.77	7.06	6.46
0.70	16.46	9.64	7.09	7.86	4.23	4.45	3.88	4.02	4.83	4.51
0.75	14.79	8.35	6.20	6.50	3.65	3.81	3.31	3.31	4.19	3.95
0.80	13.38	7.17	5.54	5.54	3.23	3.19	2.84	2.71	3.36	3.32
0.90	11.29	5.57	4.52	4.17	2.53	2.24	2.24	1.94	2.69	2.39
1.00	9.79	4.49	3.83	3.20	2.09	1.57	1.84	1.32	2.25	1.80
1.50	5.82	2.03	2.26	1.27	1.29	0.53	1.18	0.43	1.37	0.62
2.00	4.18	1.20	1.66	0.78	1.06	0.25	1.03	0.17	1.10	0.32
2.50	3.31	0.84	1.34	0.55	1.01	0.10	1	0	1.02	0.14
3.00	2.75	0.66	1.16	0.39	1	0	1	0	1	0
4.00	2.13	0.38	1.02	0.15	1	0	1	0	1	0

**Table 4:** Under LLF, ARL and SDRL results using P distribution for Bayesian-AEWMA *CC*, for $\psi$ = 0.25, *n* = *5.*

Shift	Bayes-EWMA SRS		Bayes-AEWMA SRS		Bayes-AEWMA RSS		Bayes-AEWMA MRSS		Bayes-AEWMA ERSS	
	*ARL*	SDRL	ARL	SDRL	ARL	SDRL	ARL	SDRL	ARL	SDRL
	L = 2.9050		h = 0.242		h = 0.0545		h = 0.0386		h = 0.0761	
0.00	371.05	368.88	370.14	434.88	369.55	464.59	370.37	422.06	371.08	372.83
0.20	179.81	175.30	86.77	83.25	48.19	43.15	33.89	28.36	53.96	50.53
0.30	105.54	101.21	55.44	42.26	24.06	22.33	18.67	15.89	31.14	28.66
0.40	64.00	59.50	36.76	25.98	13.92	13.40	11.09	10.41	17.42	15.66
0.50	41.56	37.30	25.86	16.88	10.59	8.53	7.22	7.16	10.81	9.71
0.60	28.54	24.33	19.65	12.16	6.08	5.59	4.80	4.76	7.06	6.07
0.70	20.96	16.82	15.62	9.17	4.38	3.66	3.45	3.22	5.65	4.32
0.75	18.11	14.02	14.23	8.29	3.88	3.09	3.04	2.74	5.68	4.42
0.80	15.89	11.94	12.83	7.30	3.50	2.70	2.68	2.22	4.52	3.15
0.90	12.61	8.89	10.79	5.90	2.87	1.90	2.20	1.64	3.68	2.23
1.00	10.27	6.77	9.25	5.00	2.48	1.47	1.87	1.18	3.18	1.72
1.50	5.18	2.50	4.95	2.80	1.55	0.64	1.20	0.43	2.02	0.75
2.00	3.46	1.33	2.97	1.81	1.19	0.40	1.03	0.18	1.53	0.54
2.50	2.64	0.85	1.97	1.13	1.04	0.21	1	0	1.03	0.12
3.00	2.19	0.62	1.48	0.73	1	0	1	0	1	0
4.00	1.66	0.50	1.09	0.30	1	0	1	0	1	0

**Table 5:** The run length profiles for Bayesian-AEWMA *CC* using PP distribution based on LLF, for $\psi$ = 0.10, *n* = *5.*

Shift	Bayes-EWMA SRS		Bayes-AEWMA SRS		Bayes-AEWMA RSS		Bayes-AEWMA MRSS		Bayes-AEWMA ERSS	
	*ARL*	SDRL	ARL	SDRL	ARL	SDRL	ARL	SDRL	ARL	SDRL
	L = 2.7018		h = 0.0856		h = 0.0411		h = 0.0365		h = 0.0449	
0.00	371.50	368.69	369.58	524.70	370.46	440.87	370.57	385.21	369.38	463.78
0.20	122.45	113.13	70.53	91.22	38.45	32.21	26.97	23.27	36.15	32.81
0.30	67.08	57.94	35.71	45.25	21.14	17.81	19.56	15.34	22.98	20.56
0.40	41.41	32.72	21.24	26.29	13.45	12.31	12.47	10.51	14.25	13.76
0.50	28.05	19.84	13.66	16.90	8.93	8.88	7.79	8.91	9.50	9.81
0.60	21.08	13.64	9.46	11.08	6.41	6.08	5.58	5.69	6.94	6.11
0.70	16.27	9.54	6.94	7.70	4.52	4.27	3.91	4.10	4.83	4.51
0.75	14.73	8.23	6.22	6.53	3.81	3.69	3.33	3.35	4.13	3.94
0.80	13.33	7.17	5.50	5.58	3.14	3.12	2.82	2.76	3.41	3.43
0.90	11.22	5.60	4.52	4.15	2.51	2.22	2.24	1.92	2.67	2.38
1.00	9.65	4.44	3.77	3.17	2.06	1.55	1.84	1.35	2.26	1.71
1.50	5.82	2.02	2.26	1.29	1.28	0.53	1.18	0.42	1.38	0.31
2.00	4.18	1.20	1.66	0.78	1.06	0.25	1.02	0.16	1.10	0.31
2.50	3.30	0.84	1.35	0.55	1.01	0.10	1	0	1.02	0.15
3.00	2.76	0.65	1.16	0.39	1	0	1	0	1	0
4.00	2.13	0.38	1.02	0.15	1	0	1	0	1	0

**Table 6:** Utilizing LLF, the run length profile using PP distribution for Suggested *CC*, for $\psi$ = 0.25, *n* = *5.*

Shift	Bayes-EWMA SRS		Bayes-AEWMA SRS		Bayes-AEWMA RSS		Bayes-AEWMA MRSS		Bayes-AEWMA ERSS	
	ARL	ARL	ARL	SDRL	ARL	SDRL	ARL	SDRL	ARL	SDRL
	*L* = 2.8986		*h* = 0.2414		*h* = 0.0547		*h* = 0.0385		*h* = 0.0764	
0.00	370.23	368.87	368.67	359.45	370.08	417.78	370.01	417.25	368.93	402.90
0.20	177.79	174.80	98.16	83.24	38.98	38.88	38.39	36.04	30.73	28.08
0.30	103.68	99.36	54.92	41.45	23.55	22.10	18.70	16.02	30.52	28.72
0.40	63.21	58.43	36.19	25.48	13.82	13.14	11.33	10.43	17.43	15.79
0.50	41.26	37.00	25.97	17.13	8.64	8.39	7.15	7.16	11.15	9.96
0.60	28.35	24.16	19.68	12.21	5.98	5.53	4.92	4.95	7.51	6.25
0.70	20.68	16.45	15.56	9.19	4.36	3.68	3.49	3.24	5.74	4.44
0.75	18.04	14.01	14.18	8.26	3.84	3.05	2.98	2.61	4.94	3.62
0.80	15.79	11.87	12.79	7.24	3.92	3.18	2.66	2.17	4.45	3.01
0.90	12.52	8.81	10.74	5.93	3.48	2.67	2.18	1.60	3.74	2.33
1.00	10.23	6.71	9.20	4.98	2.83	1.85	1.88	1.20	3.18	1.73
1.50	5.18	2.51	4.94	2.79	2.46	1.46	1.20	0.44	2.01	0.72
2.00	3.46	1.33	2.95	1.81	1.19	0.40	1.03	0.19	1.89	1.22
2.50	2.65	0.86	1.98	1.14	1.04	0.20	1	0	1.08	0.11
3.00	2.19	0.62	1.48	0.72	1	0	1	0	1	0
4.00	1.66	0.50	1.09	0.30	1	0	1	0	1	0

The findings displayed in Tables 1, 2, 3, 4, 5 and 6 regarding the Bayesian-AEWMA CC, which utilized different LFs and RSS schemes for both P and PP distribution, indicate that this method is highly effective in identifying out of control signals when contrasted to other sampling schemes analyzed in this study. Specifically, the Bayesian-AEWMA CC with MRSS stands out as the most efficient in identifying such signals. These results demonstrate the superiority of the suggested Bayesian-AEWMA CC method when it comes to identifying out-of-control signals, and its effectiveness in ensuring that prompt corrective measures can be taken.

## Real-life applications

Many analysts in the field of SPC use both actual and simulated datasets to evaluate the performance of *CC*s. In the current study, we have utilized real-dataset obtained from Montgomery^33^ to demonstrate the functioning and carrying out of the Bayesian AEWMA *CC* utilizing various ranked-based sampling designs based on P and PP distributions applying two distinct LFs. The dataset contains 45 samples, with each sample containing 5 wafers. The semiconductor production process involves photolithography in conjunction with the hard bake process, and measurements are taken in microns. Samples were collected at hourly intervals, with the first thirty samples representing in controlled process (Phase I), and the next fifteen samples representing the process that is out-of-control (Phase II). It is important to note that all observations in the Phase-II dataset have been adjusted by adding 0.017, indicating an upward shift in the core process mean.

**Figure 4:**
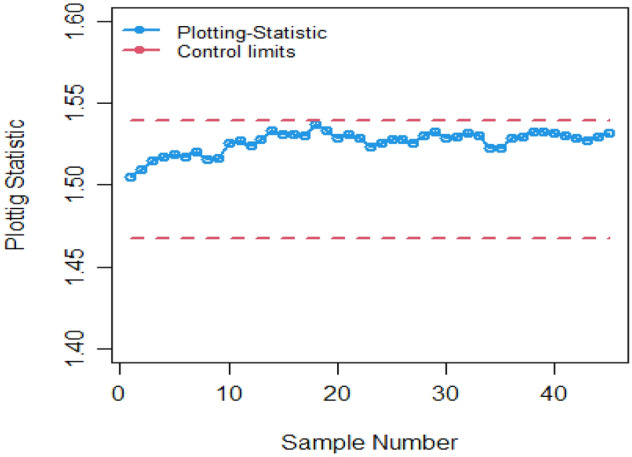
Utilizing SRS, Bayesian-EWMA *CC* using SELF.

**Figure 5:**
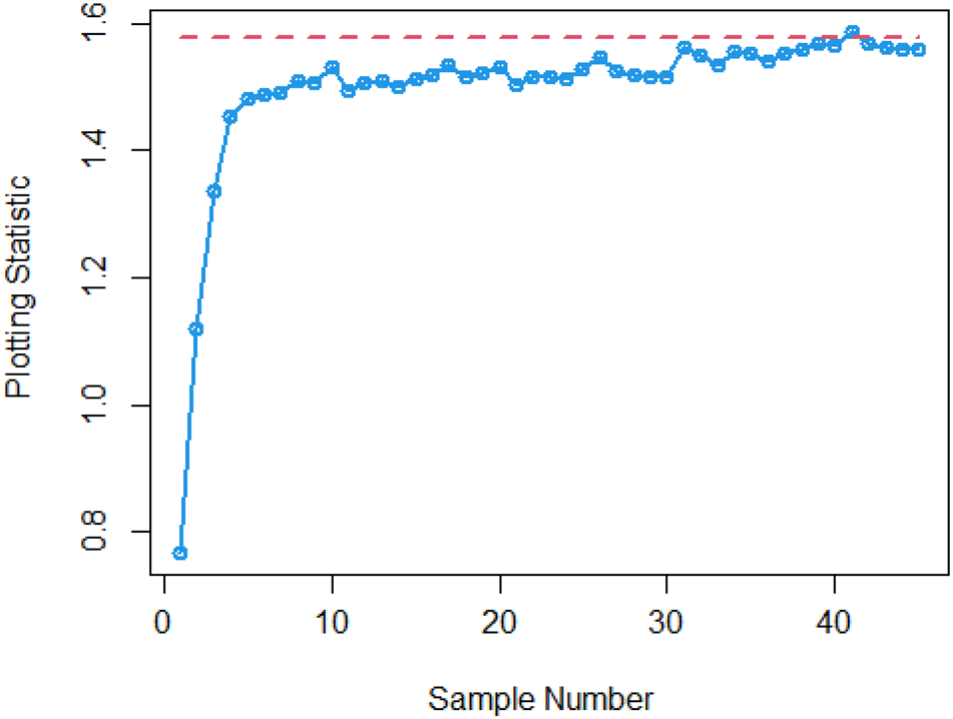
Using SRS, the Bayesian AEWMA *CC* based on SELF.

**Figure 6:**
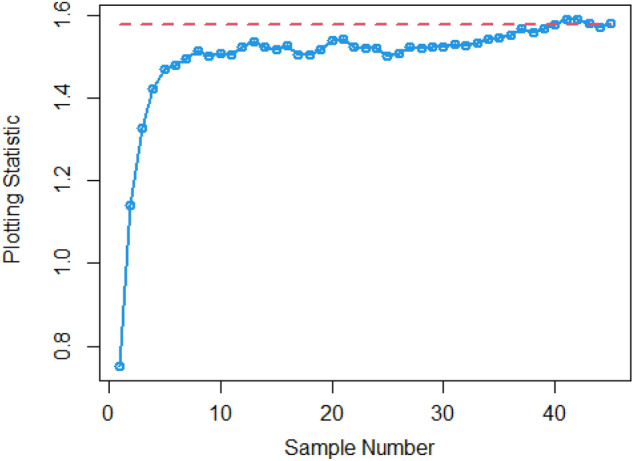
Utilizing SELF, Bayesian AEWMA *CC* based on RSS design.

**Figure 7:**
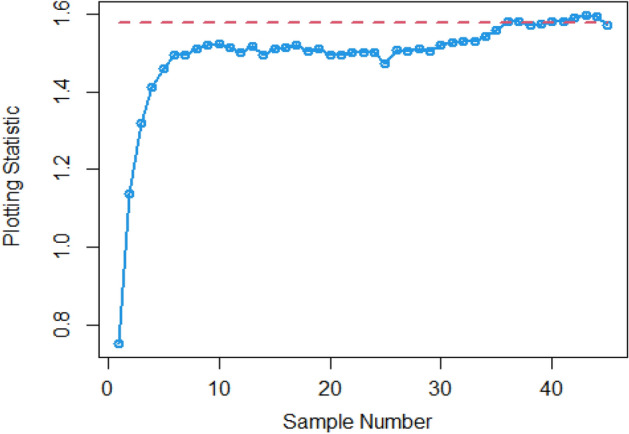
Based on SELF, the *Bayesian AEWMA CC* utilizing MRSS.

**Figure 8:**
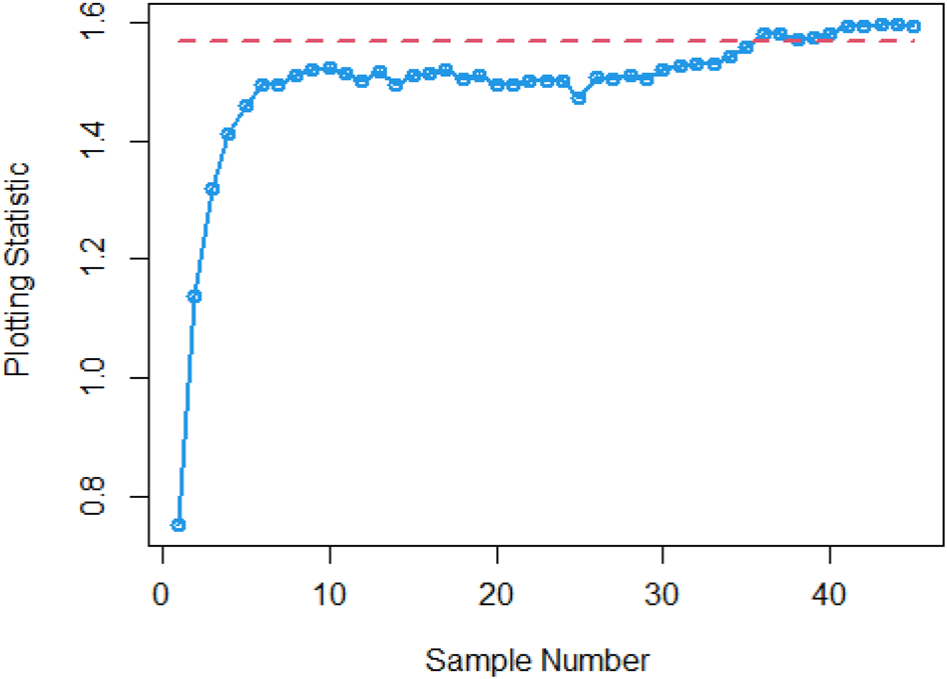
Based on ERSS, the *Bayesian-AEWMA CC* applying SELF.

Figures 4 and 5 depict the EWMA and AEWMA *CC*s utilizing Bayesian theory applying P and PP distribution based on SRS utilizing SELF. For Fig. 4, the *CC* indicates that the process cannot detect out of control signals, while for Fig. 5 the process becomes out of control on 40th sample. Figures 6, 7, and 8 display the offered Bayesian *CC* applying SELF with different RSS methods using P and PP distribution. Figures 6, 7 and 8 indicate that the process detect out of control signals for RSS on the 37th sample, for MRSS on 34th, and for ERSS on 36^th^ sample. It can be illustrate from plots 1–8, that the Bayesian-AEWMA *CC* under RSS methods, as proposed, is more effective in pointing the out-of-control signals than both the Bayesian-EWMA and Bayesian-AEWMA *CC* using SRS.

## Conclusion

This study proposes a novel Bayesian AEWMA *CC* that applies different ranked-based sampling designs under informative prior and two different LFs using P and PP distributions for the process mean. The outcomes presented in Tables 1, 2, 3, 4, 5 and 6 demonstrate the performance of the proposed *CC* applying RSS designs compared to the *Bayesian-AEWMA CC* utilizing SRS. The ARL plots (Figs. 1, 2 and 3) demonstrate the superiority of the proposed Bayesian *CC*. Additionally, to evaluate the performance of the suggested *CC* under various ranked-based sampling designs, a numerical example was applied to the hard bake process in semiconductor manufacturing. Moreover, the proposed Bayesian-AEWMA *CC* for both P and PP distributions was more effective at pointing the out-of-control signals compared to the EWMA and AEWMA *CC*s using the Bayesian approach under SRS. The Bayesian AEWMA *CC* utilizing various ranked-based sampling designs proposed in this study can be extended to other memory-type *CC*s. Additionally, it is worth noting that this approach is not restricted to normal distributions and can be adapted to accommodate data that follows a binomial distribution or Poisson. Nevertheless, to incorporate Bayesian updating, the likelihood function would require modification. Expanding this proposed technique to non-normal distributions and other types of control charts (*CC*s) could provide a more comprehensive picture of the underlying data and help detect subtle variations that may not be evident using conventional statistical methods. This could, in turn, help organizations identify potential quality concerns earlier, take corrective actions more quickly, and reduce the likelihood of costly errors and defects. For instance, in the healthcare sector, extending this approach to *CC*s could help detect anomalies in patient data, enabling healthcare providers to intervene promptly and provide timely care to patients. Similarly, this technique could be used in the finance industry to identify fraudulent activities and potential errors in financial transactions. In manufacturing, expanding this approach to non-normal distributions and other types of *CC*s could help detect variations in the production process, enabling manufacturers to improve their product quality and reduce waste.

## Data Availability

The datasets used and/or analyzed during the current study are available from the corresponding author upon reasonable request. Further, no experiments on humans and/or the use of human tissue samples involved in this study.
